# Case of Ulceration of the Larynx

**Published:** 1814-07

**Authors:** 

**Affiliations:** Apothecary to the Middlesex Hospital


*For the Medical and Physical Journal.*

*Case of Ulceration of the Larynx; by* Mr. CLAYTON, Apothecary to the Middlesex Hospital.

JAMES SMITH, a black, on the 1st of January, 1814, became my patient. He was labouring under symptoms apparently pulmonic, viz. cough, pain in the side, and shortness of breath; but neither of these was increased on inspiration. It was also observed that he possessed the power of inflating the chest completely, though not without great effort; and *no difficulty was experienced in expiration.* In addition to these symptoms, he complained of soreness of the throat, fever, and head-ach. He expectorated a large quantity of very viscid mucus, streaked occasionally with blood; and sometimes he bled at the nose. From the known fact that black men do not bear the use of the lancet to the extent generally employed in other subjects, bleeding was not had recourse to; but general antifebrile and pectoral medicines were administered, and with seeming advantage. For the space of twenty-six days he was evidently better. The fever was lessened, and the distressing difficulty of breathing in a great measure removed. On the 26th of January the symptoms returned with considerable severity, nor could he sleep for the distress he experienced in his cough and breathing. Pressure upon the external part of the larynx gave pain, as is experienced in the last stage of phthisis. On the 6th of February the difficulty of breathing was almost insupportable, and he was obliged to keep himself sitting in an erect posture. On the 7th I was called to him in the night: he was bled, with immediate

 relief relief of the symptoms, but they soon afterwards returned, and he died on the 10th.

*Appearances of the Trachea after being removed.*—No ulceration could be seen in the larynx until it was cut open, and then an ulcer as large as a sixpence was found situated immediately behind the arytenoid cartilages, and occupying that part of the membrane which covers the cricoid cartilage. The ulcer has entirely destroyed the membrane, and has also in a great degree affected the cartilage, which, though the patient was a young man, was completely ossified, and had quite the appearance of a carious bone.

Besides the inflammation caused by the ulcer in its immediate vicinity, there was a considerable degree of inflammation in the mucous membrane, which extended to the upper part of the larynx, and might be traced down into the bronchia. But this general inflammation seemed to have commenced only a very short time before death, as there were not any of those appearances which mark an inflammation of long standing; and, upon macerating the trachea a short time in water, all appearances of the inflammation vanished.

